# Analysis of Different Key Behavioral Patterns to Score in Elite Taekwondoists According to the Weight Category and Gender

**DOI:** 10.3389/fpsyg.2021.713869

**Published:** 2021-07-19

**Authors:** Cristina Menescardi, Coral Falcó, Antonio Hernández-Mendo, Verónica Morales-Sánchez

**Affiliations:** ^1^AFIPS Research Group, Department of Teaching of Musical, Visual and Corporal Expression, University of Valencia, Valencia, Spain; ^2^Department of Sport, Food and Natural Sciences, Western Norway University of Applied Sciences, Bergen, Norway; ^3^Department of Social Psychology, Social Work, Anthropology and East Asian Studies, University of Málaga, Málaga, Spain

**Keywords:** behavior, tactics, combat sport, competition group, olympians

## Abstract

Traditionally, research in taekwondo has focused on athletes' overall performance considering the entire sample of the tournament or analyzed selected bouts while ignoring behavioral differences of athletes according to their gender and weight category. Thus, the aim of this study was to analyze the behavioral patterns used to score points in the London Olympic Games according to gender and weight category [fin (FW), feather (FTW), light (LW), and heavy (HW)] of the athletes. A total of 24,940 actions were analyzed by using observational methodology, a mixed method methodology where lag sequential and polar coordinate analysis were applied. Different patterns could be seen in the actions performed prior to score between the weight categories for both genders. To score one point, females FW performed dodges, LW used indirect attacks and HW used direct attacks, posterior counterattacks, dodges and blocks. After scoring one-point, female athletes used a variety of actions, defensives like dodges (FW/LW) and blocks (LW), and offensives like simultaneous counterattacks (FW, FTW, LW) and posterior (FTW) as well as direct (FTW, HW) and indirect (FW) attacks. No patterns were found in males when scoring one-point, only LW competitors performed a sequence characterized by the performance of dodges followed by indirect attacks prior scoring while simultaneous counterattacks occurred after score one point. To score two points, similar patterns were found for all weight groups among female competitors. Females performed openings prior to scoring (LW/HW), while anticipatory (FW/FTW) and posterior counterattacks (LW) occurred after scoring, followed by cuts (FW), direct attacks (LW) and openings (HW). In males, FTW and HW used counterattacks prior to scoring (posterior and simultaneous), while FTW and LW also used counterattacks after scoring (anticipatory and simultaneous). Prior to scoring three points different patterns were found according to the weight category, while FW females used cuts and openings, LW used dodges and posterior counterattacks. After scoring three points, FW continued to use cuts, openings and posterior counterattacks while FTW used indirect attacks and HW used simultaneous counterattacks. On the contrary, similar behavior was found in males. FW and LWs used simultaneous counterattacks prior to scoring while they used defensive actions such as openings (FW) and dodges (LW) after scoring. Male FTW used also posterior counterattacks after scoring. Prior to scoring four points females reported different key behaviors. FW used anticipatory counterattacks and LW direct attacks whereas after scoring, FTW used dodges and openings. Similarities were found in males to score four points. LW and HW performed blocks prior scoring, FW and HW performed also direct attacks while FTW performed posterior counterattacks prior score. After scoring, defensives actions were performed such as dodges (LW) or cuts (FW, FTW, HW) and indirect attacks (LW, HW). This is one of the first studies analyzing behavioral patterns in taekwondo according to the weight category and gender of the athletes. The observed relationships identified different behavioral patterns according to the weight category for each gender and demonstrate the necessity to individualize trainings according to the athlete's characteristics (weight and gender). It is suggested that coaches and psychologists train athletes to improve their decision-making according to the successful patterns extracted in this study.

## Introduction

In the field of sports performance analysis, technological advances have opened opportunities to study behavioral patterns in a new way, and thereby inform how to improve and train the performance of athletes in competition (e.g., tactics, space, time, etc.) (Menescardi et al., 2020d). In combat sports, such as taekwondo, the tactic used is vital to overcome the opponent and obtain the victory of the bout, being broadly studied in different populations such as cadet (Casolino et al., 2012; Menescardi et al., 2020a), college (Falco et al., 2014; Menescardi et al., 2015) and Olympic athletes (Menescardi et al., 2019a,b, 2021).

Previous studies have identified a development in technical-tactical dynamic patterns as a response to changes in sport regulations (Moenig et al., 2012). In this sense, attacks were mostly used to score in college championships (Falco et al., 2014; Menescardi et al., 2015) and Olympic games until the 2004 Olympic Games (Kazemi et al., 2006, 2009), while this trend was reversed in the next three Olympic tournaments (2008, 2012 and 2016 Olympic Games). Here counterattacks were the predominant tactic used to score, which can be seen as a way of neutralizing the opponent's attack by breaking the rhythm of the combat (Kazemi et al., 2010; Cular et al., 2011; Menescardi et al., 2019b, 2020b,c). Several counterattacking patterns were found, the predominant approach was the use of simultaneous and posterior counterattacks while anticipation was the least counterattacking type used (Menescardi et al., 2019b). In-depth analyses of the kicking score identified a mix of scoring patterns (Menescardi et al., 2021). One point (to the chest protector) was mainly scored with direct attacks and anticipatory counterattacks, two points (to the chest protector with turning kicks) were scored with simultaneous counterattacks, three points (to the head) with indirect attacks and anticipatory counterattacks and indirect attacks and simultaneous counterattacks for scoring four points (to the head with turning kicks).

Other relevant actions in any tactical sequence (Menescardi et al., 2020d) are defensive ones (e.g., cuts, dodges, blocks), which are used to neutralize the opponent's attacks and counterattacks. Defensive actions could suppose a mean of 64.7 (SD = 21.4) actions per bout as observed in World Cups and Championship competitions (González-Prado et al., 2015). Moreover, in the Olympic Games in London 2012 it was observed that athletes used dodges to avoid opponents' attacking and counterattacking actions as well cuts and finally blocks to avoid being kicked (Menescardi et al., 2019c). Further, kicking patterns could be affected by whether the athletes are winning or losing the bout. Clear patterns were seen through six World Cups of 2000–2008 (González-Prado et al., 2015), where losing competitors tried to minimize the score difference by attacking more, while winning athletes tended to maintain the score difference with a more defensive behavior. Further, analysis of defensive actions used by the athletes in the different weight categories showed that light categories performed more dodges than the heavier ones, with fin competitors using the fewest. This could be because the lighter categories, except fin, have less body mass and can react quickly to an attack (Menescardi, 2016). Thus, not only offensive but also defensive actions should be considered in the analysis of behavioral sequence in combat sports such taekwondo.

Despite the influence of these factors on taekwondo bout success, there are few comparative studies regarding the performance of athletes based on their weight category (Falcó et al., 2012; Menescardi et al., 2012, 2020c). These studies revealed different behavioral patterns according to the weight category of the athletes with lower weight categories performing more actions than heavier ones. The bout outcome is also related to the tactics applied (Menescardi et al., 2020c), where fly and feather female winners performed more anticipated and posterior counterattacks than heavies who performed more cuts and posterior counterattacks than light weight category. For lighter categories the anticipation is related to the winning of the bout while being defensive is related to the winning in heavies. With regard to males, more one-point and two-point actions were performed by fly and feather in comparison with light and heavy weight categories as well as more direct actions were performed by fly than heavies who preferred to use more indirect actions (Menescardi et al., 2020c). The use of defensive and indirect actions is congruent with previous systematic review reporting more non-fighting phases (characterized by observation and low intensity actions) in heavier than lighter weigh categories (Santos et al., 2020).

In order to provide more precise information to coaches it is important to analyze the competitors' behavioral patterns (technical and tactical) based on their competition weight category (Falcó et al., 2012; Menescardi et al., 2012, 2020c). For that reason, the aim of the present study was to analyze the behavioral patterns used to score points in an Olympic Games tournament according to the gender and weight category (fin, feather, light and heavy) of the athletes.

## Methodology

### Participants

A total of 76 female and 75 male bouts at an Olympic tournament (London 2012) was recorded and analyzed across four weight categories. Only one male bout was not performed due to injury of one competitor. In taekwondo the four weight categories are distributed as follows: fin [<49 kg for females and <58 kg for males, (FW)], feather [>49 and <57 kg for females and >58 and <68 for males, (FTW)], light [>57 and <67 kg for females and >68 and <80 kg for males, (LW)] and heavy [+67 kg for females and +80 kg for males, (HW)].

A total of 24,940 actions were performed in the mentioned bouts. Since the analyzed videotapes, on which public behavior can be observed, are in the public domain, it is not necessary to acquire informed consent from the athletes concerned (American Psychological Association, 2002). The study protocol was approved by the Human Research Ethics Committee at the first author's university.

### Measures and Instruments

For codifying tactical actions in taekwondo, the taekwondo observational tool (TKDOT) tactical criteria validated by Menescardi et al. (2017) was used (Table 1). HOISAN 1.5.6 software was used for recording and coding the data (Hernández-Mendo et al., 2012), including all of the constituting criteria of the observational tool. With regard to computerized encoding, the data were codified as multi-events as they have been proposed in the context of a multidimensional design. This software also provides information of the duration of actions performed.

**Table 1:** Criteria, categories, codes and categorical core of the observational tool used.

**Criteria**	**Categories**	**Code**	**Categorical core or description**
Tactics	Direct attack	DIA	Offensive action with the objective of scoring, ending with an impact on the opponent but without previous movement
	Indirect attack	INA	Offensive action in order to score, ending with an impact on the opponent and with previous movement such as a step, skip, opening, guard change, kicking trajectory modification, etc.
	Anticipated counterattack	ACA	Action that starts during the opponent's attack with the purpose of scoring. The athlete kicks the attacker during the preparatory phase (guard) and/or initial phase (when the opponent's knee is being raised)
	Simultaneous counterattack	SCA	Action that starts at the same time as the opponent's attack and has a scoring purpose. The athlete kicks at the same time as the opponent. Thus, the counter attacker kicks at the end of the attacker's initial phase (leg raised) or during the impact momentum (impact phase) of the attacker's kick
	Posterior counterattack	PCA	Action that begins after the opponent's attack (during the descending phase, or when attacker's leg touches the ground) with a scoring purpose. Athletes kick at the same time. This action (sometimes) includes a previous backward displacement to dodge the opponent's attack
	Opening	OPE	Movement to control the distance with the opponent or bridge the gap between both competitors
	Block	BLO	Defensive actions to avoid the impact of a kick by placing one arm or leg between the protector and the leg of the opponent. This does not have a scoring objective
	Dodge	DOD	Defensive actions to avoid the impact of a kick by placing one arm or leg between the protector and the leg of the opponent. This does not have a scoring objective
	Cut	CUT	Defensive forward movement to avoid being beaten by a close opponent, and to prevent the attacking action from being completed. This does not have a scoring objective
Score	1 point	SC1	Effective action performed on the protector with linear, circular or punch techniques that scores 1 point
	2 points	SC2	Effective action performed on the protector with a previous spin technique that scores 2 points
	3 points	SC3	Effective action performed on the head with linear or circular technique or to the chest protector with a spinning technique that scores 3 points
	4 points	SC4	Effective action performed on the head with a technique with previous spin that scores 4 points

### Design and Procedures

The current observational study followed a design characterized as follows (Anguera and Hernández-Mendo, 2013): follow-up between sessions—Olympic championship; nomothetic—focusing on 128 athletes; and multidimensional—analysis of two criteria: tactical actions used and their effectiveness. A procedure was developed to train the observers (Menescardi et al., 2017, 2020b). Six observers, divided into two groups (A and B), were involved in the reliability analysis of the data. To evaluate the inter-observer reliability, each observer analyzed six combats. To evaluate the intra-observer reliability, one of the observers analyzed the same six combats twice. Cohen's kappa (κ) was used to calculate intra- and inter-observer reliability, whose results showed Cohen's κ values above 0.85, showing almost perfect conformity (Landis and Koch, 1977; López-López et al., 2015).

### Statistical Analysis

To discover the most effective actions, the adjusted residuals (*z*) were used to indicate whether the difference between the frequency observed and the frequency expected was statistically significant (≥|1.96| implies a 95% confidence interval). When *z* ≥ 1.96 shows an excitatory relationship, this means that it contributes significantly to the effectiveness of this action related to the criterion.

After determining effective actions of one- (SC1), two- (SC2), three- (SC3), and four-points (SC4), lag sequential analyses, followed by polar coordinate analyses of the tactical actions used to score, were performed using HOISAN software and drawn with Matlab (Perea et al., 2012; Menescardi et al., 2019a). Previous studies have applied both statistical analysis due to its complementarity in observational studies (Castañer et al., 2017). Lag sequential analysis allows researchers take into consideration both prospective and retrospective perspectives (i.e., actions that follow and precede the score) in order to construct sequences and detect when a technical-tactical element facilitate the occurrence of a given score (retrospective). On the contrary, the prospective perspective is based on considering those elements as posterior to the scoring action (*z* > 1.96; *p* < 0.05). The number of negative (−1 and −2) and positive lags (+1 and +2) were used as suggested by previous studies (Anguera et al., 2018). Polar coordinate analysis used *z* from lag sequential analysis to calculate *Zsum* statistics (Zsum = Pz/√n) (Cochran, 1954). This process presents a polar coordinate map that shows the associations between the behavior of interest or focal behavior (scoring actions) and the conditional behaviors (technical-tactical behaviors). Thereafter, the relationships are shown in four different quadrants depending on their angle and the strength of the relationship (Arias-Pujol and Anguera, 2017):

- **Quadrant I (0°-90°)**. Indicates that the focal and conditional behaviors are mutually activated in both perspectives. That is, the conditioned behaviors occur before and after the focal behavior (+, +).
- **Quadrant II (90°-180°)**. Indicates that the focal behavior inhibits the conditional behaviors but is also activated by them. That is, the conditioned behavior precedes but does not follow the focal behavior (+, –).
- **Quadrant III (180°-270°)**. Indicates that the focal and conditional behaviors are mutually inhibited. That is, the conditioned behavior neither precedes nor follows the focal behavior (–, –).
- **Quadrant IV (270°-360°)**. Indicates that the focal behavior activates the conditional behaviors but is also inhibited by them. That is, the conditioned behavior does not precede but follows the focal behavior (–, +).

In this work, only relationships with a module or radium length (*r*) of the vector > 1.96 are considered significant (*p* < 0.05) and included in the results. A total of four lag sequential and polar coordinates analyses (i.e., one point, two points, three points, and four points) were conducted for each weight category according to their sex, in accordance with the aims of the study.

## Results

### Results for Females

**Figure 1:**
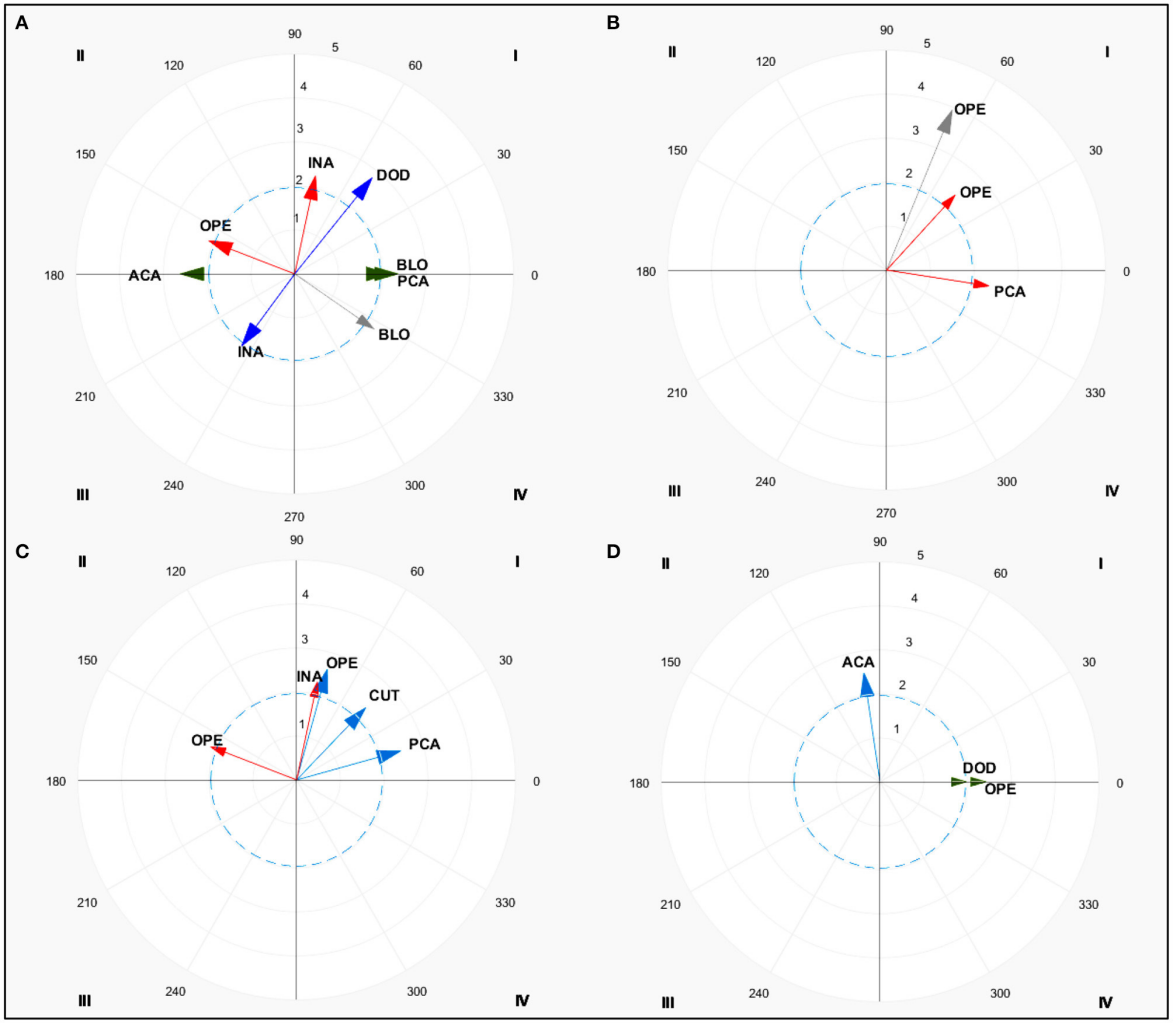
Polar coordinate analysis of one-point **(A)**, two-point **(B)**, three-point **(C)**, and four-point **(D)** actions for females. Color arrows indicate significant relationships (*z* > 1.96) in each weight category: fin (blue), feather (green), light (red), and gray (heavy). DIA, Direct Attack; INA, Indirect Attack; BLO, Blocks; DOD, Dodge; OPE, Opening; ACA, Anticipatory counterattack; SCA, Simultaneous counterattack; PCA, Posterior counterattack.

One point was mostly scored by FW and FTW throughout direct (*z* = 5.3 and *z* = 4.1, respectively) attacks. FTW also obtained one point with the use of anticipatory (*z* =6.3) and simultaneous (*z* = 2.1) counterattacks. LW mainly used direct attacks (*z* = 5.3) and posterior counterattack (*z* = 3.1) while HW used direct attacks (*z* = 4.6) and simultaneous counterattacks (*z* = 4.6). Two points were scored by FW with anticipatory (*z* = 5.1) counterattacks while FTW and HW used simultaneous counterattacks (*z* = 4.5 and *z* = 4.6, respectively). LW weight scored two points by performing indirect attacks (*z* = 2.2). Three points were predominantly scored by FW with anticipatory counterattacks (*z* = 3.7) and indirect attacks (*z* = 3.9) while FTW used indirect attacks (*z* = 3.3), and posterior (*z* = 2.2) and simultaneous (*z* = 2.5) counterattacks. LW used direct and indirect attacks (*z* = 4.5 and *z* = 3.3, respectively) to score three points while HW used direct attacks and anticipatory counterattacks (*z* = 3.2 and *z* = 4.9, respectively) to score three points. Four points were scored largely with posterior counterattacks by FW (*z* = 2.0) and simultaneous counterattacks by FTW and HW (*z* = 2.1 and *z* = 3.4, respectively). Different patterns could be seen in the actions performed to score between weight categories. Lag sequential analyses is shown in Table 2 while polar coordinate analyses for females are shown in Figure 1 and Table 3.

**Table 2:** Positive relationships between tactical actions and effectiveness in retrospective and prospective perspective for females.

		**Retrospective**		**Prospective**	
**Effectiveness**	**WC**	**R-2**	**R-1**	**R+1**	**R+2**
1 point	Fin	DOD (3.09)	-	DOD (2.36)^*^	INA (2.30)
				SCA (2.27)^*^	
	Feather	-	-	BLO (2.18)	DIA (3.05)
				SCA (3.92)^†^^*^	
				PCA (2.82)	
	Light	-	INA (3.15)	DOD (3.43)^†^	–
				SCA (2.56)^†^	
	Heavy	DIA (2.02)	BLO (2.10)	DIA (2.86)	–
			DOD (2.40)		
			PCA (2.30)		
2 points	Fin	–	–	ACA (2.51)^‡^	CUT (2.69)
	Feather	–	–	ACA (3.07)^†^	–
	Light	–	OPE (2.41)^¥^	PCA (3.65)	DIA (2.79)
	Heavy	–	OPE (5.15)^*^	–	OPE (2.49)
3 points	Fin	CUT (2.32)	–	CUT (3.07)	OPE (3.18)
		OPE (3.56)		PCA (2.30)	
	Feather	–	–	–	INA (2.78)
	Light	–	DOD (2.94)	–	–
			PCA (2.70)		
	Heavy	–	–	SCA (3.02)	–
4 points	Fin	ACA (3.51)	–	–	–
	Feather	–	–	DOD (3.35)	OPE (3.90)
	Light	–	DIA (2.05)	–	–
	Heavy	–	–	–	–

*Only significant patterns are shown*.

*WC, Weight category; DIA, Direct Attack; INA, Indirect Attack; BLO, Blocks; DOD, Dodge; OPE, Opening; ACA, Anticipatory counterattack; SCA, Simultaneous counterattack; PCA, Posterior counterattack*.

† *Similar behavior found in the lag to fin;*

‡ *Similar to feather;*

* *Similar to light;*

¥ *Similar to heavy weight category in the same effectiveness*.

**Table 3:** Polar coordinate significant patterns for effective actions in females.

**Effectiveness**	**WC**	**Category**	**Q**	**Radium**	**Angle**
1 point	Fin	DOD	I	2.81	51.03
		INA	III	2.03	233.49
	Feather	BLO	I	2.18	0.00
		ACA	III	2.62	180.00
		PCA	I	2.36	0.00
	Light	OPE	II	2.11	158.95
		INA	I	2.28	77.99
	Heavy	BLO	IV	2.20	325.34
2 points	Light	OPE	I	2.31	47.48^¥^
		PCA	IV	2.37	351.24
	Heavy	OPE	I	3.94	67.63^*^
3 points	Fin	PCA	I	2.47	15.47
		CUT	I	2.28	46.13
		OPE	I	2.61	74.46
	Light	OPE	II	2.11	158.95
		INA	I	2.28	77.99
4 points	Fin	ACA	II	2.51	98.43
	Feather	DOD	I	1.98	0.00
		OPE	I	2.43	0.00

*WC, Weight category; DIA, Direct Attack; INA, Indirect Attack; BLO, Blocks; DOD, Dodge; OPE, Opening; ACA, Anticipatory counterattack; SCA, Simultaneous counterattack; PCA, Posterior counterattack*.

* *Similar to light;*

¥ *Similar to heavy weight category in the same effectiveness*.

### Results for Males

**Figure 2:**
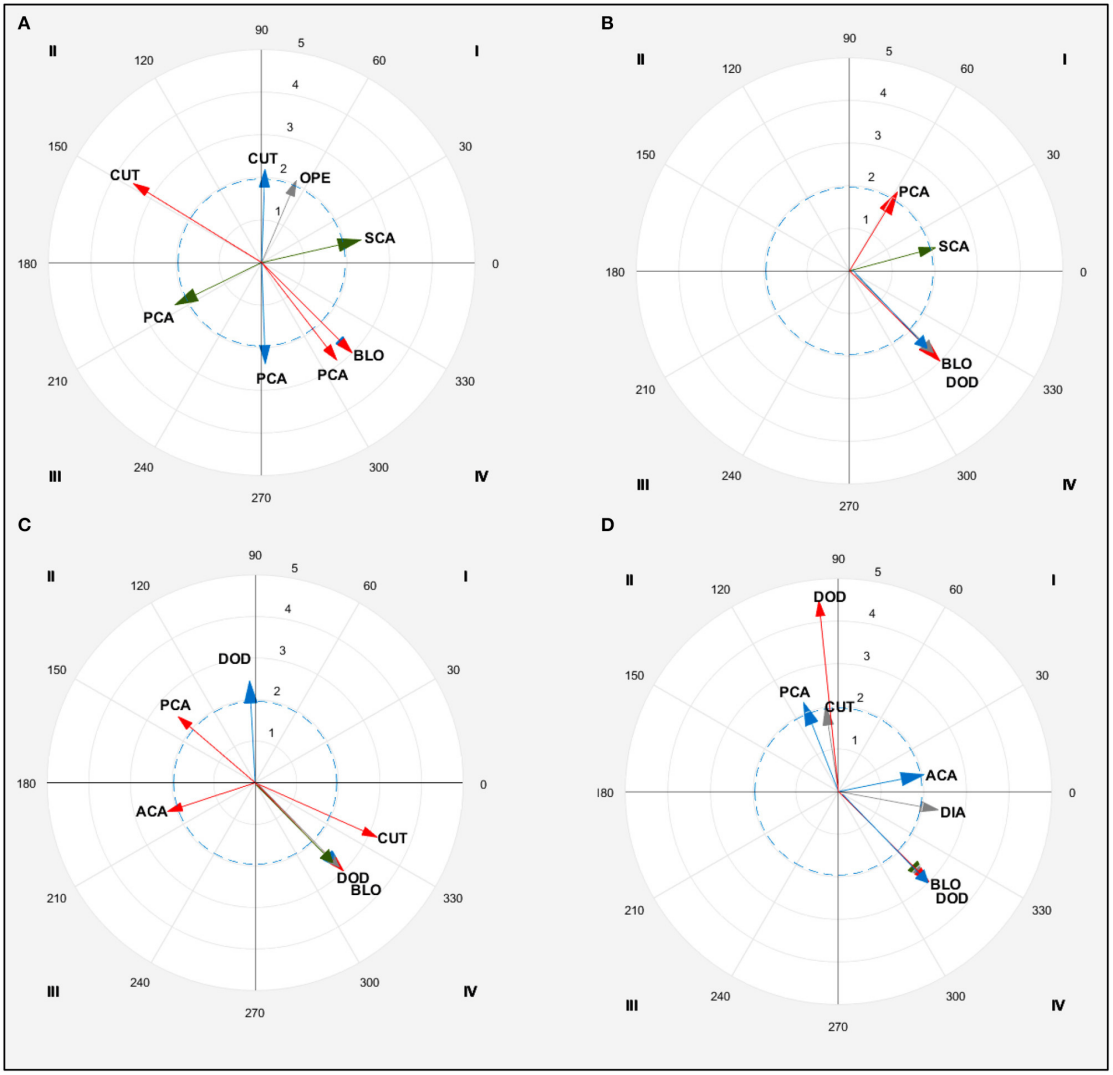
Polar coordinate analysis of one-point **(A)**, two-point **(B)**, three-point **(C)**, and four-point **(D)** actions for males. Color arrows indicate significant relationships (*z* > 1.96) in each weight category: fin (blue), feather (green), light (red), and gray (heavy). DIA, Direct Attack; INA, Indirect Attack; BLO, Blocks; DOD, Dodge; OPE, Opening; ACA, Anticipatory counterattack; SCA, Simultaneous counterattack; PCA, Posterior counterattack.

One point was mostly scored by FW and FTW males with direct (*z* = 4.0 and 3.1, respectively) and indirect attacks (*z* =2.6 and 2.0, respectively) and anticipatory counterattacks (*z* = 3.4 and 5.1 respectively). FW also scored one point with simultaneous counterattacks (*z* = 3.2). LW competitors scored only with attacks (direct, *z* = 3.2; indirect, *z* = 3.9). HW scored with direct (*z* = 3.9), anticipatory (*z* = 3.2) and posterior counterattacks (*z* = 6.2). Two points were scored by FW with indirect actions (*z* = 4.0), FTW with indirect (*z* = 2.1) and simultaneous (*z* = 3.5) actions and HW mainly with simultaneous actions (*z* = 3.3). Three points were predominantly scored by FW with anticipatory and simultaneous counterattacks (*z* = 2.0). FTW scored three points with direct and anticipatory actions (*z* = 3.5 and 2.8, respectively) while HW used anticipatory and simultaneous counterattacks to score (*z* = 4.9 and 2.2 respectively). LW competitors scored with indirect attacks (*z* = 4.1). Four points were scored largely with posterior counterattacks by FW (*z* = 2.0), indirect attacks by LW (*z* = 3.7) and simultaneous counterattacks by HW (*z* = 2.5). Different patterns could be seen in the actions performed to score between weight categories. Lag sequential analyses is shown in Table 4 while polar coordinate analyses are shown in Figure 2 and Table 5.

**Table 4:** Positive relationships between tactical actions and effectiveness in retrospective and prospective perspective for males.

		**Retrospective**		**Prospective**	
**Effectiveness**	**WC**	**R-2**	**R-1**	**R+1**	**R+2**
1 point	Fin	–	–	–	–
	Feather	–	–	–	–
	Light	DOD (1.97)	INA (1.97)	SCA (2.14)	–
	Heavy	–	–	–	–
2 points	Fin	–	–	–	–
	Feather	PCA (2.20)	–	ACA (3.21)	–
	Light	–	–	–	SCA (2.45)
	Heavy	SCA (3.19)	–	–	–
3 points	Fin	BLO (2.24)	–	OPE (2.59)	–
		SCA (1.97)^*^			
	Feather	–	–	–	PCA (4.14)
	Light	SCA (2.20)^†^	–	DOD (2.22)	–
	Heavy	–	–	–	–
4 points	Fin	–	–	–	CUT (2.70)^‡^
	Feather	DIA (2.56)	–	–	CUT (2.30)^†^
	Light	BLO (3.63)^¥^	BLO (2.70)	DOD (2.07)	INA (2.14)
			PCA (3.08)		
	Heavy	BLO (3.11)^*^	DIA (2.21)	AIN (2.6)	CUT (3.77)

*Only significant patterns are shown*.

*WC, Weight category; DIA, Direct Attack; INA, Indirect Attack; BLO, Blocks; DOD, Dodge; OPE, Opening; ACA, Anticipatory counterattack; SCA, Simultaneous counterattack; PCA, Posterior counterattack*.

† *Similar behavior found in the lag to fin;*

‡ *Similar to feather;*

* *Similar to light;*

¥ *Similar to heavy weight category in the same effectiveness*.

**Table 5:** Polar coordinate significant patterns for effective actions in males.

**Effectiveness**	**WC**	**Category**	**Q**	**Radium**	**Angle**
1 point	Fin	BLO	IV	3.00	315.00^*^^¥^
		CUT	I	2.19	87.97
		PCA	IV	2.38	272.05^*^
	Feather	SCA	I	2.39	12.84
		PCA	III	2.27	206.09
	Light	BLO	IV	3.00	315.00^†^^¥^
		CUT	II	3.53	148.35
		PCA	IV	2.89	307.54^†^
	Heavy	BLO	IV	3.00	315.00^†^^*^
		OPE	I	2.09	67.16
2 points	Fin	BLO	IV	3.00	315.00^‡^^*^
	Feather	BLO	IV	3.00	315.00^†^^*^
		SCA	I	2.09	15.07
	Light	BLO	IV	3.00	315.00^†^^‡^
		PCA	I	2.17	58.75
	Heavy	DOD	IV	3.00	315.00
3 points	Fin	BLO	IV	3.00	315.00^‡^^*^
		DOD	II	2.46	93.13
	Feather	BLO	IV	3.00	315.00^†^^*^
	Light	BLO	IV	3.00	315.00^†^^‡^
		CUT	IV	3.21	335.92
		ACA	III	2.22	198.20
		PCA	II	2.44	139.34
	Heavy	DOD	IV	3.00	315.00
4 points	Fin	BLO	IV	3.00	315.00^‡^
		ACA	I	2.05	11.15
		PCA	II	2.25	111.40
	Feather	BLO	IV	3.00	315.00^†^
	Heavy	DOD	IV	3.00	315.00
		CUT	II	2.02	98.67
		DIA	IV	2.39	349.59

*WC, Weight category; DIA, Direct Attack; INA, Indirect Attack; BLO, Blocks; DOD, Dodge; OPE, Opening; ACA, Anticipatory counterattack; SCA, Simultaneous counterattack; PCA, Posterior counterattack*.

† *Similar to fin;*

‡ *Similar to feather;*

* *Similar to light;*

¥ *Similar to heavy weight category in the same effectiveness*.

## Discussion

To the best of the authors' knowledge this is the first study identifying the most effective actions and behavioral patterns of taekwondo competitors according to their weight category and sex. The major relevance of the current study was the effective actions and significant patterns extracted which can help coaches and psychologists to prepare athlete's for future competitions.

### Female Behavior According to Their Weight Category and Effective Actions

To score one-point, similar patterns were found between the four weight categories. That is, the use of direct attacks followed by simultaneous counterattacks emerged as one of the most used patterns. As previously reported in elite competition (Menescardi et al., 2019b), both actions allow athletes to react within a short period of time (Falco et al., 2014). Despite their extensive use in competition and their proven effectiveness, it should be noted that sometimes the opponent has not enough space or skills to perform defensive actions to defend themselves from the opponent's action. For one-point score, it should be noted the tactical repertoire mastered (Menescardi et al., 2020d) by FTW female athletes as they did not only score with attacks but also with counterattacks (anticipatory and simultaneous). Additionally, LW females used posterior counterattacks (to an indirect action) to score one point. This is in line with findings from previous studies (Menescardi et al., 2019c) following the ‘if-then’ statements based on stimulus and responses (Falco et al., 2014). The use of posterior and indirect actions by FTW and LW competitors, prior to and after scoring, supports their tactical repertoire mastery to continue the tactical schema initiated by their opponents. As taekwondo is a combat sport where actions can be enchained by competitors (De la Fuente García and Castejón, 2016; Menescardi et al., 2020c), and where mastering offensive and defensive actions remain essential. Defensive actions, such as dodges and blocks, performed by FW and FTW, which were performed prior to and after scoring, as well as openings by LW which were performed prior to scoring, are revealed as essential to avoid being scored against by the opponents. In this sense, lighter weight competitors (i.e., FW and FTW) opt to face the opponent (through dodges and blocks which required a close combat/clench) while heavier weights (i.e., LW) opt to gain distance from the opponent and re-structure their strategy. This explanation is plausible according to previous studies (Vasconcelos and Del Vecchio, 2021) noting the different effort: pause ratio of wushu sanda heavy competitors (1.6:1) in comparison to other weight categories (ranging from 2.6:1 to 2.9:1). Likewise, the more static behavior of HW categories has also been pointed out in the current study.

To score two points a similar pattern was found, characterized by the performance of counterattacks (anticipatory for FW, and simultaneous for FTW and HW) to respond to opponent's openings. That resulted in effective actions (Menescardi et al., 2019b), except for LW who used indirect attacks to score (which were counterattacked with posterior counterattacks). The LW category can be characterized by an approach where they mostly meet their opponent through indirect actions. They choose the correct moment to initiate the attack, revealed as an effective characteristic of elite athletes (Chiodo et al., 2012), which could explain why indirect actions are effective, while posterior counterattacks are not always effective, if the attack is not properly feinted and the attacker hits the target area (Borysiuk and Waskiewicz, 2008).

To score three points, similar patterns were found for the different weight categories regarding the use of attacks and counterattacks (Menescardi et al., 2019c). FWs used anticipatory and indirect attacks to score, which were defensed or posteriorly counterattacked, following the pattern found to score one point (Falco et al., 2014). FTWs used indirect attacks and simultaneous counterattacks while attacks (direct or indirect) were used by LWs in response to defensive actions and posterior counterattacks used prior to scoring. HW competitors used direct attacks to score, which were not properly simultaneously counterattacked. They also used anticipatory actions to score three points. Thus, attacks are mainly related to scoring three points as also found in other studies (such as Lee Dae Hoon; Menescardi et al., 2019c).

Finally, to score four points, the use of anticipatory actions prior to scoring was found in FW competitors while defensive actions such as dodges and openings were used after scoring in FTW competitors. The scarce use of four- point actions (Lystad et al., 2020; Menescardi et al., 2020b) is also highlighted in this study which could explain the lack of difference across the weight categories. Despite the lack of sequences found, it should be noticed the use of counterattacks (simultaneous and posterior) to score by the different weight categories. It seems that elite athletes tended to observe their opponent and execute counterattacks to the head to achieve four-points as they manage to surprise the opposite and then score (López-López et al., 2015).

### Male Behavior According to Their Weight Category and Effective Actions

To score one point, it is surprising the scarce relationships found with weight categories, acknowledging the highly variable behavior of competitors. It is well-known that the more variable the athlete's behavior is, the more reaction time is required by the opponent to analyse the number of possible stimulus–response options to choose the appropriate response (Borysiuk and Waskiewicz, 2008; Kwok, 2012). Further, mastering the tactical repertoire, as occurred in the lighter weight categories in females, are important characteristic of elite athletes. According to the female competitors' results, LW male competitors used attacks followed by simultaneous counterattacks, in line with previous studies (Menescardi et al., 2019b). The use of simultaneous counterattacks seems the main option chosen to respond to opponent's direct attack as it is expected to stop the opponent's advance and break the rhythm of the combat. The fact that both kicks were performed at the same time and legs crash made the attacker lose their balance. Equal pattern was also found between FW and LW male competitors, who used cuts prior to and after scoring as well as posterior counterattacks and blocks after scoring, highlighting the relevance of a structured defense in every moment of the bout. On the contrary, FTW were characterized by use of simultaneous counterattacks and HW by use openings, pointing out their more static form of competing over other weights categories, as previous studies have suggested (Santos et al., 2020).

To score two points, in FTW males a pattern characterized by indirect attacks and anticipatory counterattacks was found. In this sense, attackers optimized their offensive action to score. Despite previous studies reporting the effectiveness of anticipatory actions (López-López et al., 2015), these kinds of actions should be performed in the right time to score and surprise the opponent (Falco et al., 2014; Menescardi et al., 2015). This could explain the effectiveness of simultaneous actions for the two-point scores observed in the present study. Our results are in line with previous studies that reported athletes' persistence to perform counterattacking actions (such as posterior by LWs and simultaneous by FTWs) to score by performing turning kicks (i.e., the only type of kick that allows this score; López-López et al., 2015). Defensive behaviors (blocks and dodges) were found after scoring, and performed by all competitors (except FWs). The use of attacks, counterattacks and defensive actions in a sequence support the relevance to master all possible tactical schemes to respond to opponent's actions.

To score three points, an offensive pattern characterized by counterattacking the opponent (anticipatory or simultaneously) was found in FW competitors. In this weight category, being offensive by kicking to the head is an effective strategy when the opponent does not use a defensive action in an appropriate manner. Similarly, the use of indirect attacks is used by LW competitors when they observe an opponent that does not dodge in an appropriate manner. For that reason, the use of the first rounds of the bout to study and test the technical-tactical behavior of the opponent remains essential (González-Prado et al., 2015). Moreover, athletes should explore the opponent's responses to different attacks (e.g., giving or cutting distance, or performing a counterattack) (Franchini et al., 2008) and, from the point of view of the opponent, to observe the characteristics of the attack (i.e., short or long) (Menescardi et al., 2019b). Athlete's behavior was also similar across weight categories after scoring by performing defensive actions (i.e., blocks by FW, FTW, and LW, and dodges by HW). This could be because lighter (FW, FTW, and LW) categories opt to adopt a close combat position, which requires a quick response to opponent's action, while HWs prefer to increase the distance with the opponent to re-structure their bout strategy, as occurred in females. Taekwondo athletes spend time in well-defined distances from their opponent, and especially HW competitors (Vasconcelos and Del Vecchio, 2021), who can rest and perceive how to better reach the target and score while controlling also the distance from the opponent. The major use of defensive style by heavier competitors are in line with prior studies (Menescardi et al., 2019b, 2020c), reporting that HWs used less blocks and preferred to perform less risky actions than those requiring less time to respond to the opponent (as it could be expected by blocks and cuts performed by lighter weight competitors who need to be continuously moving forward, invading the opponent and compete over a shorter distance). This is also in line with previous studies (Engwerda and Lidor, 2020), which found differences in pace, and explosivity, and sport performance in judo athletes of different weight categories.

To score four points, in line with previous results (Menescardi et al., 2019b), LW competitors took advantage of when their opponent was in a close combat (clench) after a block and posterior counterattack to initiate a new (indirect) attack which was not well dodged by the opponent. The continuation of the sequence (also known as counter-counterattacks, Fargas, 1990) supposed to the opponent a more complex stimulus identification and response selection (Kwok, 2012), ending in a scored point. On the contrary, HWs preferred to perform counterattacks (simultaneous) to score, continuing the sequence with a cut. The shorter sequence performed by heavier competitors is congruent with previous judo studies (Kuvačić et al., 2017), showing lighter weight competitors outscored their heavier peers in muscular endurance, jumping ability, and balance ability. Finally, all competitors performed defensive actions after scoring (blocks, cuts or dodges), preventing kicks from longer distances (Lystad et al., 2020). The behavior of FW competitors was characterized by performing counterattacking actions (anticipatory prior and after score, and posterior prior) and HW competitors used direct attacks after scoring. Once again it can be observed the more direct patterns chosen by HW category in comparison with longer (and indirect) actions initiated by lighter weights (González-Prado et al., 2015; Santos et al., 2020).

Additionally, it should be noted that the scoring behaviors for each weight category are independent of the athletes' sex (e.g., the use of attacks and simultaneous counterattacks by LW competitors, the use of anticipatory counterattacks to score two and three-points as well the use of posterior ones to score four-points by FW athletes). Thus, it is suggested to organize the training together attending building on their similar characteristics in competition. On the contrary, when a different behavioral pattern is observed, for different weight categories and sex, it is necessary to individualize trainings according to the athlete's characteristics (weight and gender). Lastly, it might be interesting that coaches and psychologists train athletes to improve their decision-making according to the successful patterns extracted in this study.

This study is not absent of limitations. It should be acknowledged that athletes' behavior to score was not analyzed according to the round of the bout and/or championship, which could further explain the effectiveness of the actions and whether the behavior shown varies depending according to the round or phase of the championship. Likewise, the presentation of the results of the 2012 Olympic Games could also be considered a limitation, since in 2016 a more current Olympic championship has been held. Despite the mentioned limitation it should be noted that observational studies of Olympic taekwondo are scarce given the complexity and detail of this type of study, as well as the high volume of data obtained and aspects to be reviewed in each bout. Therefore, future studies are necessary to confirm whether these trends are maintained with the new regulations. Further studies including not only polar coordinates and lag sequential analysis but also T-Pattern analyses should be conducted for a better understanding of performance in this sport. Despite these limitations, the results of the current study provide a better understanding of technical-tactical behavior used to score according to the athletes' weight category and gender in Olympic taekwondo bouts and constitute a basis for future research in this sport.

From a practical point of view, coaches should design technical-tactical strategies to physically train actions that permit scoring (QI/QII) according to the athletes' sex and weight category according to their bout strategy, as well as psychologist should help athletes to train psychological aspects such as attitude, confidence, motivation and stress control. To do this, mental training protocols of the technical-tactical visualization to enhance strengths and exploit the opponent's weak points are suggested. In addition, simulate training methods to mechanize and automatize tactical scenarios and coping strategies for stress through or self-regulation work to make good decisions (‘if-then’ sequences) as well as relaxation work, imagery, attention and concentration training, self-instruction or positive reinforcement can be used to produce adaptive behaviors for motor improvement, helping athletes reach their maximum sports performance.

## Conclusion

Taekwondo coaches, athletes and researches could also use the observational methodology described in the current study to highlight the most important factors that affect the effectiveness during competition, and train the preceding actions used to score (as triggers of behavior of interest: scoring point/s).

One point was mostly scored by direct attacks in every weight category independently of their gender. Athletes also used other techniques to score by surprising their opponents according to their categories and sex, such as FW and FTW males who used indirect and anticipatory actions. FW males as well as FTW and HW females also used simultaneous counterattacks while LW females and HW males used posterior counterattacks and LW males also indirect and anticipatory attacks. To score one point, females used attacks (indirectly by LW and directly by HW) as well as defensive actions (dodges by FW and HW competitors and blocks by HW). After scoring, female athletes used defensive actions (dodges and blocks), counterattacks (simultaneous and posterior) and attacks (direct and indirect). In males, LW competitors performed dodges and indirect attacks prior to scoring while simultaneous counterattacks occurred after that.

Two points were scored by counterattacking actions (anticipated by FW females and simultaneous by FTW and HW competitors) as well as indirect actions by LW females, and FW and FTW males. Prior to scoring two points, all weight female competitors performed openings, followed by counterattacks (anticipatory and posterior), and thereafter defensive actions (cuts and openings) and direct attacks. In males, FTW and HW used counterattacks prior to scoring (posterior and simultaneous), while FTW and LWs also used counterattacks after scoring (anticipatory and simultaneous).

Three points were predominantly scored by anticipatory (FW competitors, FTW males, HW competitors) and indirect actions (FW, FTW and LW competitors), as well as FTW females used posterior and simultaneous actions. LW females and HW competitors also used direct attacks. FW and HW males also used simultaneous counterattacks. Prior to scoring three points, FW and LW females used defensive actions (cuts, openings and dodges) and posterior counterattacks (only LWs). After scoring, FW females continued using defensive actions and posterior counterattacks while FTW females used indirect attacks (and posterior counterattacks) and HW used simultaneous counterattacks. Male FW and LW competitors used blocks and simultaneous counterattacks prior to scoring while they used openings and dodges after scoring.

Four points were scored largely with posterior actions by FW competitors, simultaneous by FTW and HW competitors and indirect attack by LW males. Prior to scoring four points, FW males used anticipatory counterattacks and LW direct attacks whereas after scoring, FTW used dodges and openings. FTW females used direct attacks prior to scoring four points while LW and HW also used blocks and posterior counterattack. After scoring, they used defensive actions (dodges and cuts) in addition to indirect attacks.

## Data Availability Statement

The raw data supporting the conclusions of this article will be made available by the authors, without undue reservation.

## Author Contributions

CM, CF, and AH-M conceived the study, participated in its design and coordination, contributed to video coding, data collection, conducted statistical analyses and contributed to the interpretation of the results, drafted the manuscript, and approved the final manuscript as submitted. VM-S participated in the study design, contributed to the interpretation of the results, reviewed and provided feedback to the manuscript, and approved the final manuscript as submitted. All authors made substantial contributions to the final manuscript.

## Conflict of Interest

The authors declare that the research was conducted in the absence of any commercial or financial relationships that could be construed as a potential conflict of interest.
